# Impact of virtual reality immersion on exercise performance and perceptions in young, middle-aged and older adults

**DOI:** 10.1371/journal.pone.0307683

**Published:** 2024-10-30

**Authors:** Angela Hibbs, Gavin Tempest, Florentina Hettinga, Gillian Barry

**Affiliations:** Department of Sport, Exercise and Rehabilitation, Faculty of Health and Life Sciences, Northumbria University, Newcastle, United Kingdom; The Hong Kong Polytechnic University, HONG KONG

## Abstract

This study compared the effect of high and low levels of virtual reality (VR) immersion during moderate and high intensity cycling-exercise in younger (18–35 years), middle-aged (36–50 years), and older (51–69 years) adults. Thirty participants (5 female and 5 males per age group) completed moderate (steady state: 15 minutes at 60–75% maximum heart rate (MHR)) and high (sprint: 10 x 30 second sprints at 75–85% MHR) intensity cycling in four conditions: VR using a head-mounted display (High HMD), room-projector (Low Room), screen-projector (Low Screen) and No VR. Exercise performance measures (cadence, distance, power output) were recorded. Following each VR condition, exercise enjoyment (PACES) and exercise engagement (Flow State Scale) were measured. Results showed that exercise intensity had a significant effect of age on; heart rate (steady state and sprint), cadence (sprint) and distance (sprint) (p’s <0.05). A significant effect of condition was observed during the sprint exercise for heart rate (p < 0.05). No other significant condition effects were observed for exercise performance. Significant effects of condition were identified for exercise engagement relating to sense of control (p < 0.01) and loss of self-consciousness (p < 0.05) with the highest values occurring in the Low Screen condition for all age groups, while transformation of time was significantly different (p < 0.05) for the middle-aged adults (highest during High VR and Low Screen). These results indicate that irrespective of age, participants found themselves in control and immersed more during the Low Screen VR condition than the other VR and No VR conditions. The findings indicate that VR immersion impacts exercise performance and exercise engagement in different age-groups and therefore should be considered when using VR to promote exercise behaviour.

## Introduction

Physical inactivity is a global health problem and puts many healthcare organisations under great strain. It is well-known that engaging in physical activity through exercise decreases the risk of musculoskeletal, metabolic, cardiovascular and neurological conditions [1, 2]. Yet, one-third of adults over the age of 18 do not meet the recommended physical activity guidelines [3]. Barriers towards physical activity include environmental context and social influences [4]. For example, finding the motivation and confidence to attend exercise sessions outside of the home can be an overwhelming barrier due to time, transport, safety and/or financial concerns. Recently, largely due to the COVID-19 global pandemic, there is a greater availability of home-based exercise options such as static cycling trainers and advances in virtual reality (VR) training software [5] (e.g. Zwift, wireless head-mounted displays; HMD). Therefore, exercising at home has become increasingly popular and more achievable allowing to engage a wider community of exercisers [6–8]. For many adults, VR equipment/software may be an appealing way to increase exercise engagement and enjoyment [9] and subsequently help individuals fulfil their recommended physical activity levels.

In a review of exercise and VR studies, Mouatt et al. [10] provided evidence to support a positive effect of VR on exercise motivation, enjoyment, affect and engagement. Mouatt et al. [10] reported that home-based exercise is accessible and an appealing option for adults and that exercise using VR can alter an individuals’ perception of the activity. For example, increases in enjoyment are believed to be due to the level of distraction VR provides away from negative perceptions (i.e., feeling like it is too difficult). In turn, some individuals report less perceived effort when using VR compared to no VR, potentially enabling individuals to exercise for longer or increase the likelihood that they would repeat the activity in the future [11]. In line with the theory of attentional focus [12], VR alters the internal and external focus of attention during activity whereby attention is drawn towards external factors (i.e., the environmental and social context provided by VR and away from internal factors (i.e., physiological challenges), resulting in a positive affective state [11, 13]. Interacting with external, social or environmental factors (such as, exercising outdoors or with others) is important for exercise regulation [14]. Therefore, VR offers a platform to improve the exercise experience and examine the mechanisms of which environmental and social cues impact exercise regulation through the manipulations of external factors in controlled settings.

While there is support for use of VR to promote home-based exercise and to understand (external) factors driving exercise regulation, there is a further need to examine how well VR mimics reality (i.e., level of immersion) and how the VR immersion alters exercise perception. Participants have reported that more immersive VR (e.g., HMDs) is more beneficial (e.g., led to higher enjoyment and engagement) than low immersive VR (i.e., projected images) or exercise without VR [10, 15, 16]. The level of VR immersion is understood to create different levels of presence (or engagement in the activity), for example, the level of presence is higher using HMDs than using a screen projector [17]. A higher level of presence correlates with an increase in exercise enjoyment and adherence [13, 17–19]. What contributes to presence, or engagement, is outlined in the Flow State Scale theory [20]. Flow is achieved by reduced task difficulty, increased self-efficacy and control, and changes in awareness (from internal to external factors) [21]. The Flow State Scale has previously been used in exergaming (exercise plus gaming) research which showed that those in the exergaming exercise groups felt more focused on the task, had clear goals and felt immersed in the activity [22, 23].

Although evidence supports improved exercise enjoyment and engagement using VR, participants have reported complications using high immersive VR which include ‘cyber-sickness’ and discomfort from wearing HMDs [16, 24]. Such reports may deter some participants from using VR. Mouatt et al. [10] call for further research to provide definite recommendations for which VR platforms (i.e., HMD, large or small screen-projectors) best influence specific exercise outcomes (e.g., enjoyment and engagement) and are appropriate for specific populations based on health status and age in particular. The combination of exercise and VR may be a way for adults to overcome some of the barriers to physical activity engagement, however previous studies tend to focus on younger adults (majority <40 years old; Mouatt et al. [10]. Van Schaik et al. [25] and Zelinski and Reyes [26] reported positive findings (increased distraction and presence) in older adults during VR based exercise and highlighted its potential to improve cognitive abilities. Yet, no comparison of the effects of different levels of VR based cycling-exercise on performance, enjoyment and engagement (i.e., flow) in different ages groups has been conducted to date.

The aim of this study was to examine the effects of cycling-exercise at two different intensities beneficial for health (moderate steady state and high intensity sprint training (HIT)) under different levels of VR immersion (High HMD, Low Room (projector), Low Screen, and No VR) on exercise performance and perceptions (exercise enjoyment and engagement (including flow state)) in younger, middle-aged, and older adults. We hypothesised that during cycling exercise in the VR environment (low and high immersion), exercise performance (e.g., a higher cadence, power output and/or distance) and exercise perception (e.g., lower perceived effort, higher exercise enjoyment and engagement) would be different to No VR in all age groups and that exercising in a VR environment would drive attentional focus towards external factors, and away from internal factors. We also expect to observe some age group preferences in terms of which VR environment is preferred. By establishing the effects of different VR immersion on exercise performance and perceptions while exercising at different target exercise intensities, these findings will inform the development of age-appropriate exercise regimes incorporating VR technology.

## Methods

### Participants

Thirty participants (male = 15, female = 15; see Table 1) were recruited from the University and local community (between June 2021 and December 2021) and assigned to one of three age groups (18–35, 36–50 and 51–69 years; male = 5, female = 5 in each age group). All participants reported being physically active (>30minutes of moderate activity 5 times per week) and reported no current musculoskeletal injuries. Previous experience of VR or cycling on a static trainer was not monitored. The study procedure was reviewed and approved by the Ethics Committee at Northumbria University (submission reference number: 32856). All participants provided written informed consent prior to participation in the study and the study was conducted conforming to the Declaration of Helsinki. The study design was non-blind with the experimenters knowing the identity of the participants.

**Table 1:** Participant information for the three age groups (Mean±SD).

Age Group (years)	n	Age (years)	Height (cm)	Body mass (kg)	Resting Heart Rate (bpm)
18–35	10	25.4±5.64	169.81±10.36	67.37±13.07	80.36±9.82
36–50	9*	40.6±4.72	169.82±9.31	70.61±11.64	77.63±7.78
51–69	10	66.1±1.52	170.02±8.55	75.57±11.02	77.78±8.20

*One participant in the 36–50 group was unable to complete the four conditions therefore their data were excluded from further analysis.

### Procedure

The participants completed two visits to the laboratory (2–14 days apart). On arrival for the second visit, participants confirmed that they were of the same state of health and mind as their first visit. On the first visit, participants provided their informed consent, and their resting heart rate was measured using a chest strap (Garmin, Hampshire, UK) and computer (Garmin Edge 530). Participants maximum heart rate (MHR: 220-age) and heart rate zones corresponding to moderate (60% MHR) and high (75–85% MHR) intensities were calculated. Participants then completed a cycling exercise protocol in two of the four experimental conditions (the remaining conditions were completed on their second visit). Between conditions, the participants sat and rested until heart rate returned to <10 beats per min of their resting heart rate. During the resting period, participants completed questionnaires indicating their exercise enjoyment and engagement related to the recently completed exercise condition. The order of conditions across sessions was randomised between all participants.

### Cycling exercise protocol

**Fig 1:**
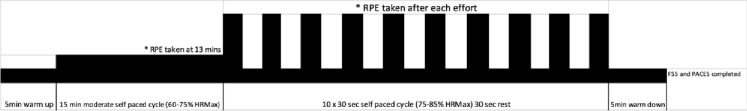
The cycling-exercise protocol used for each condition. RPE = rating of perceived exertion. PACES = physical activity enjoyment scale. FSS = Flow state scale (engagement). HRMax = maximum heart rate.

During the experimental conditions, participants cycled on a road bike (Pinnacle Lithium 3) positioned on a SMART indoor cycling trainer (Tacx Flux 2). Saddle height was adjusted for each participant and heart rate was monitored using a Garmin (Garmin, Hampshire, UK) chest strap with data collected on a cycling computer (Garmin Edge 530). Power output (W; average), cadence (RPM; average) and distance covered (m; total) were recorded. Participants completed a self-paced 5-min warm up, 15-mins steady state moderate intensity exercise (60–75% MHR) and 10 30second high-intensity sprints (75–85% MHR, 30second rest in-between). Rating of perceived exertion (RPE; Borg Scale [27]) was used to provide a measure of perceived effort (i.e., how hard the exercise felt) during the exercise protocol and was recorded at the end of the steady state exercise (minute 13) and following each of the 10 30second sprints. Following the completion of each VR condition, participants completed the PACES questionnaire (to measure perceived enjoyment following exercise [28]). A higher PACES score reflected a greater level of enjoyment. Participants also completed the Flow State Scale questionnaire [29] (used as a measure of perceived exercise engagement [30]). The scale has been well reported in previous studies to be effective in reflecting engagement level during exercise [30, 31]. The Flow State Scale comprises nine subcategories: challenge–skill balance, action/awareness merging, clear goals, unambiguous feedback, intense concentration, control over the task at hand, loss of self-consciousness, transformation of time, and autotelic experience. An overview of the exercise protocol can be seen in Fig 1.

### Experimental (virtual reality) conditions

**Fig 2:**
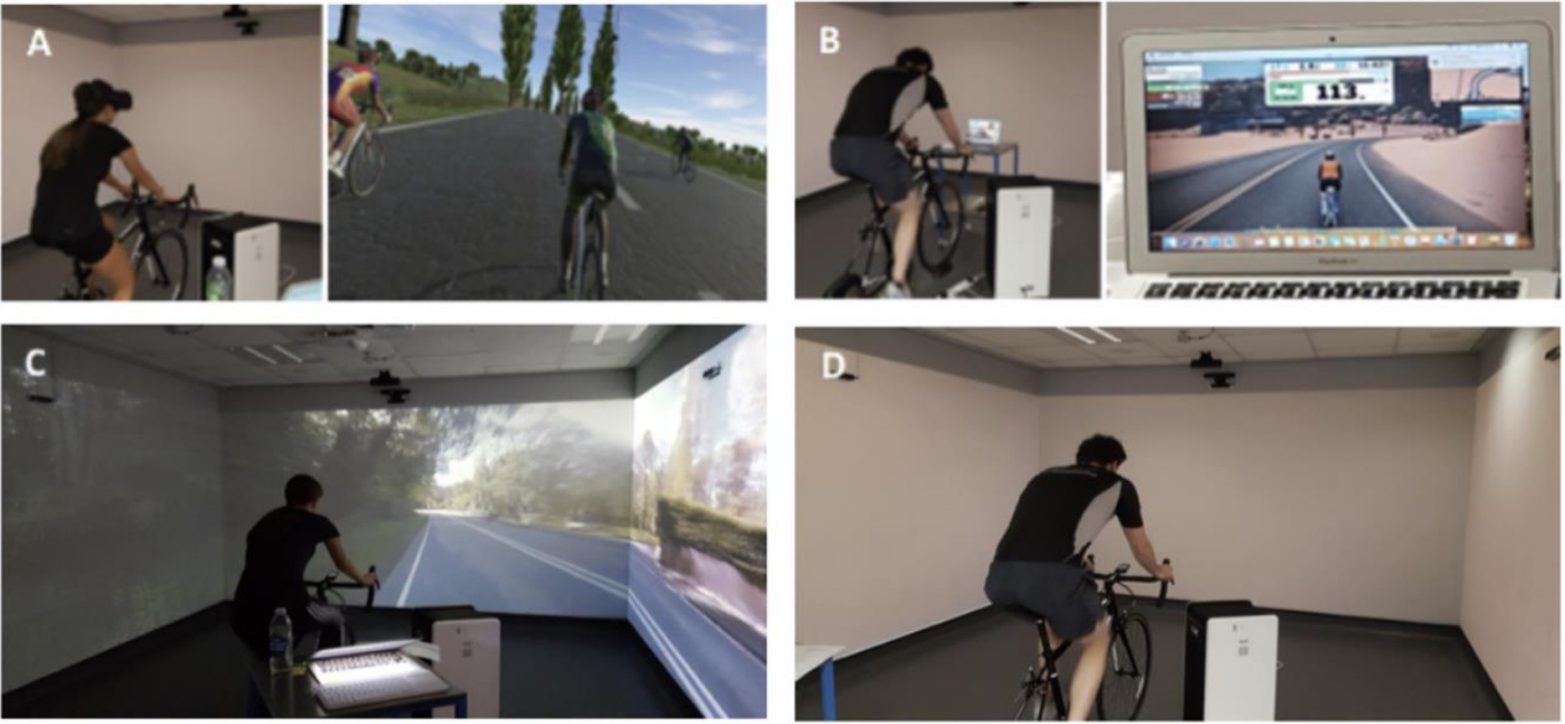
The four experimental conditions: (A) High HMD VR, (B) Low Screen-projection VR, (C) Low Room-projection VR, and (D) No VR.

The VR conditions were graded from low to high in terms of visual immersion according to Rakowski and Gruber [32]. Low immersive environments include “a computer-generated three-dimensional virtual space experienced through standard audio-visual equipment, such as a desktop computer with a two-dimensional monitor, whereas high immersive environments involving a computer-generated 360° virtual space that can be perceived as being spatially realistic, due to the high immersion afforded by a head-mounted device” [32]. In the high VR condition, participants wore a HMD displaying a virtual track (Oculus Quest 2 (Model number MH-B) Headset with VZ Play software). The headset eye resolution was 1440x1600 with a 90Hz refresh rate and a brightness level set at 80%. The headgear and SMART trainer responded to movement and cycling effort, respectively (i.e., the perspective of the VR environment changed when the participant moved their head (Fig 2A). In the Low Screen condition, participants viewed a laptop monitor (MacBook Air, model A1466, resolution rate 1440x900) positioned in front of the exercise bike and trainer (approximately 1m away) (Fig 2B; Zwift [33]. Laptop settings were the same for all participants with a brightness level of 80% and a screen refresh rate of 60Hz. The SMART trainer responded to cycling effort (i.e., changes in the participants’ power output and cadence). In the Low Room condition, participants watched a video of an outdoor cycle ride projected via 3 projectors (Sony VPL-PHZ61; resolution rate 1920x1200) onto 3 walls (sides and front) of a purpose-built immersive suite (Immersive Interactive, V3.1.6–0; Fig 2C). The cyclist was positioned 2metres away from the wall projection. The projection brightness level was set at 80% with no other room lighting on. The video was recorded from the cyclist’s perspective as the cyclist navigated along country roads and was not linked to cycling effort. Finally, a No VR condition was included whereby participants completed the cycling protocol only (Fig 2D) with no technology present.

### Statistical analysis

One participant in the 36–50 years group experienced nausea and dizziness during the High VR immersion condition and could not complete the protocol, therefore, their data were excluded from the analysis. No other participant reported sufficient levels of sickness to abort the experimental protocol or raised significant concerns regarding VR sickness. SPSS (V27) was used for all statistical data analysis. Data were checked for normality. A series of two-way (Age*Condition) ANOVAs with repeated measures were conducted for (i) height, body mass and resting heart rate (manipulation check), heart rate (average), exercise performance variables (cadence (average), power output (average), total distance) and RPE recorded during 15-min (moderate intensity) steady state (mean) and (high intensity) sprints (mean of the 10 30s sprints), and (ii) perceptual responses: exercise enjoyment (PACES) and engagement (for each sub-scale of the Flow State Scale). Significant (p < 0.05) main and interaction effects were followed-up with pairwise comparisons with Bonferroni correction or simple effects tests, respectively. Mean and standard deviations are reported (unless specified).

## Results

### Manipulation check

The participant characteristics are reported in Table 1. No significant main effects of height, body mass or resting heart rate were observed for the three age groups (p’s > 0.05).

### Exercise performance

#### Steady state cycling performance

*Heart rate (average)*. A significant main effect of Age, F(2,26) = 12.786, p = .001, ES = .05, showed that heart rate (bpm) was significantly higher for the younger age group (18–35, M = 133, SD = 11) compared to the middle (36–50, M = 122, SD = 13) and older (51–69, M = 112, SD = 13) age groups (p’s < 0.05). No further main or interaction effects were recorded (p’s > 0.05). No significant main or interaction effects were recorded for cadence, power output or distance (p’s > 0.05; see Table 2).

**Table 2:** Steady state cycling performance and rating of perceived exertion (RPE) for each condition (No VR, Low Screen VR, Low Room VR, High VR) by age group.

Variable	Condition	Age Group (years) (mean±SD)		
		18–35	36–50	51–69
Heart Rate^a^ (bpm)	No VR	132.5±7.55	127.22±11.11	114.80±12.86
	Low Screen VR	134.30±16.74	118.00±10.83	116.00±12.06
	Low Room VR	133.6±10.56	123.56±14.18	107.70±13.89
	High VR	131.10±8.17	119.11±12.99	110.90±13.93
Cadence (rpm)	No VR	66.00±15.83	71.56±10.01	67.90±8.39
	Low Screen VR	69.00±12.28	73.56±14.78	60.40±13.30
	Low Room VR	65.10±16.09	71.89±11.25	62.40±8.75
	High VR	64.20±14.04	73.11±11.84	64.10±8.94
Distance (m)	No VR	4583.00±1126.01	5377.78±1046.75	4589.70±1268.59
	Low Screen VR	5141 00±1138.18	5489.11±1029.75	4558.10±1409.62
	Low Room VR	5088.00±979.24	5417.78±1139.77	3983.20±1159.79
	High VR	4807.00±1163.49	5022.22±1112.42	4218.40±1289.67
Power (w)	No VR	74.10±32.30	94.89±26.34	76.70±28.09
	Low Screen VR	81.00±22.21	97.78±29.11	78.10±29.95
	Low Room VR	83.1±28.30	90.22±20.07	67.00±27.52
	High VR	76.90±33.51	85.11±23.39	66.90±25.33
RPE	No VR	11.50±1.78	11.33±2.65	12.70±1.06
	Low Screen VR	11.10±1.52	11.44±1.01	11.90±1.66
	Low Room VR	11.10±1.52	10.56±2.40	11.20±1.87
	High VR	11.20±2.04	11.44±1.59	11.40±2.22

^a =^ Significant main effect of age (younger adults vs. middle- and older-adults (p < 0.05).

#### Sprint cycling performance

*Heart rate (average)*. A significant main effect of Condition, F (3,78) = 3.545, p = 0.018, ES = .12, showed that heart rate (bpm) was lower during High VR (M = 140, SD = 17) compared to No VR (M = 145, SD = 15), Low Screen VR (M = 146, SD = 15) and Low Room VR (M = 141, SD = 19) (p’s < 0.05). A significant main effect of Age, F (2,26) = 39.221, p = 0.001, ES = .21, showed that heart rate was higher for the younger age group (M = 158, SD = 8) compared to the middle (M = 144, SD = 11), and older (M = 127, SD = 11) age groups (p’s < 0.05). No significant interaction was recorded (p > 0.05).

*Cadence*. A significant main effect of age, F (2,26) = 5.68, p = 0.014, ES = .28, showed that cadence (rpm) was lower in the older age group (M = 70, SD = 12) than in the younger (M = 81, SD = 12) and middle (M = 84, SD = 11) age groups (p’s < 0.05). No further main or interaction effects were recorded (p’s > 0.05).

*Distance*. A significant main effect of age, F (2,26) = 3.527, p = 0.044, ES = .21, showed that distance (m) covered was lower in the older age group (M = 186, SD = 55) than in the younger (M = 215, SD = 46) and middle (M = 237, SD = 37) age groups (p’s < .05). No further main or interaction effects were recorded (p’s > .05). No significant main or interaction effects were recorded for power output (p’s > 0.05; see Table 3).

**Table 3:** Sprint cycling performance results and rating of perceived exertion (RPE) for each condition (No VR, Low Screen VR, Low Room VR, High VR) by age group.

Variable	Condition	Age Group (years)(mean±SD)		
		18–35	36–50	51–69
Heart Rate^a^^c^ (bpm)	No VR	158.77±4.90	147.93±8.68	128.37±6.28
	Low Screen VR	158.03±13.04	148.34±9.66	130.46±11.87
	Low Room VR	159.43±8.67	141.34±13.21	123.41±12.46
	High VR	156.31±6.20	139.89±13.54	124.53±10.84
Cadence^b^ (rpm)	No VR	83.85±10.42	83.23±12.72	73.39±13.93
	Low Screen VR	81.33±14.32	85.71±10.54	68.24±13.91
	Low Room VR	79.65±15.84	80.68±11.29	69.58±12.79
	High VR	79.76±6.87	86.72±9.92	70.77±8.52
Distance^b^ (m)	No VR	214.38±43.14	229.17±41.85	186.58±51.96
	Low Screen VR	226.56±50.18	238.19±30.18	190.54±52.28
	Low Room VR	224.79±40.76	235.68±40.37	178.88±61.47
	High VR	213.75±40.44	245.33±45.73	186.90±62.60
Power (w)	No VR	154.33±56.82	158.16±36.43	117.49±55.04
	Low Screen VR	139.46±50.21	172.46±48.83	120.90±45.32
	Low Room VR	153.74±58.33	162.94±40.37	114.73±58.70
	High VR	136.90±52.94	149.54±34.68	114.91±59.81
RPE	No VR	14.30±2.38	14.54±3.01	14.63±1.12
	Low Screen VR	13.93±1.39	14.83±1.65	14.20±1.51
	Low Room VR	14.22±1.62	13.84±2.44	13.59±1.18
	High VR	13.93±1.96	14.47±2.06	13.49±1.38

Significant main effect of age

(^a^ = younger vs. middle-aged and older adults

^b^ = older vs. young- and middle-aged adults). Significant main effect of condition

(^c^ = high VR vs. No VR and Low screen VR) (p < 0.05).

### Exercise perception (RPE, enjoyment and engagement)

#### Rating of perceived exertion

No significant main or interaction effects were recorded for RPE during steady state (see Table 2) or sprint (see Table 3) cycling exercise (p’s > 0.05; see Table 3).

#### Exercise enjoyment

No significant main or interaction effects were recorded for exercise enjoyment (p > 0.05, see Table 4).

**Table 4:** Physical activity enjoyment scale scores for each condition (No VR, Low screen VR, Low Room VR, High VR) by age group.

Condition	Age Group (years)			Total
	18–35	36–50	51–69	
No VR	76.70±13.93	87.50±18.40	74.30±18.71	79.50±17.55
Low Screen VR	84.00±14.40	85.70±25.06	83.80±20.05	84.50±19.62
Low Room VR	85.80±15.40	84.10±17.36	88.40±12.65	86.10±14.82
High VR	94.90±16.72	82.11±22.52	83.10±16.14	86.86±18.81

Higher scores reflect a greater level of exercise enjoyment (Mean±SD)

#### Exercise engagement

Significant main effects of condition for sense of control, F (3, 78) = 5.65, p < 0.001, ES = 0.43 and loss of self-consciousness, F (3,78) = 2.43, p < 0.05, ES = 0.28, were recorded. For both sense of control and loss of self-consciousness mean scores were the highest in the Low Screen VR condition. Main effects of condition for both action awareness and concentration of task were close to statistical significance, F (3,78) = 3.44, p = 0.07, ES = 0.24, and F (3,78) = 2.92 p = 0.06, ES = 0.26, respectively (see Table 5).

**Table 5:** Flow state scores (exercise engagement) for the nine sub-scales for each experimental condition (No VR, Low Screen VR, Low Room VR, High VR).

Sub-Scale	Condition	Age Group (years) (mean±SD)			
		18–35	36–50	51–69	Total
Challenge skill balance (CSB)	No VR	3.93±0.89	3.81±0.6	3.9±0.72	3.87±0.73
	Low Screen VR	3.98±0.55	3.67±0.81	3.78±0.85	3.81±0.72
	Low Room VR	3.7±0.77	3.86±0.65	3.85±0.98	3.8±0.79
	High VR	3.93±0.83	3.42±1.39	3.83±0.75	3.73±0.99
Actions and awareness (AA)	No VR	3.73±0.53	3.89±0.6	3.7±0.74	3.76±0.61
	Low Screen VR	4±0.65	3.58±1.02	3.53±0.85	3.7±0.84
	Low Room VR	3.6±0.94	3.69±0.41	3.58±1.03	3.62±0.82
	High VR	3.65±1.14	2.61±1.48	3.18±1.05	3.16±1.25
Clear goals (CG)	No VR	3.73±1.08	4.11±0.5	4.08±0.68	3.96±0.79
	Low Screen VR	4.05±0.63	3.92±0.66	4.03±0.88	4±0.71
	Low Room VR	3.63±1.04	3.94±0.67	3.8±0.89	3.78±0.86
	High VR	4±0.71	3.58±1.21	3.9±0.84	3.83±0.91
Unambiguous feedback (UF)	No VR	3.78±0.72	3.72±0.76	3.83±1	3.77±0.81
	Low Screen VR	3.64±0.2	3.58±1.02	3.93±0.99	3.72±0.8
	Low Room VR	3.4±0.74	3.72±0.62	3.65±0.99	3.58±0.78
	High VR	3.68±0.99	3.75±1.02	3.43±1.05	3.61±0.99
Concentration on task (CT)	No VR	3.13±1.24	3.56±0.75	3.53±1.09	3.39±1.03
	Low Screen VR	3.9±0.94	3.47±1.11	3.63±0.38	3.67±0.84
	Low Room VR	3.88±0.73	3.78±0.95	3.68±0.84	3.77±0.81
	High VR	4.53±0.88	3.56±1.64	4.25±0.53	4.12±1.12
Sense of control (CN)^a^	No VR	4±0.73	3.89±0.91	3.98±0.7	3.95±0.75
	Low Screen VR	4.23±0.58	4.03±0.63	3.95±0.84	4.06±0.68^a^
	Low Room VR	3.63±1.06	3.92±0.54	3.25±1.29	3.58±1.02
	High VR	3.93±1.11	2.53±1.22	3.43±0.85	3.31±1.16
Loss of self consciousness (SC)^a^	No VR	4.3±0.7	4.19±0.6	4.05±0.75	4.18±0.67
	Low Screen VR	4.18±0.75	4.44±0.58	4.18±0.8	4.25±0.7^a^
	Low Room VR	4.03±0.92	3.94±0.8	3.85±0.97	3.93±0.87
	High VR	4.3±0.98	3.39±1.44	3.85±0.85	3.86±1.12
Transformation of time^b^ (TT)	No VR	3.08±0.79	2.72±0.83	3.15±0.7	2.99±0.76
	Low Screen VR	2.65±0.59	3.94±0.67	3.23±0.62	3.25±0.8
	Low Room VR	2.98±0.89	2.61±0.84	3.25±0.82	2.95±0.85
	High VR	3.2±0.99	3.47±1.01	3.08±0.74	3.24±0.9
Autotelic experience (AE)	No VR	3.33±0.71	3.61±0.59	3.15±0.97	3.35±0.77
	Low Screen VR	3.58±0.58	3.31±1.01	3.3±0.94	3.39±0.83
	Low Room VR	3.68±0.69	3.61±0.83	2.95±0.87	3.4±0.84
	High VR	3.95±0.98	3.14±1.51	3.33±1.11	3.48±1.21

^a^ Significant main effect for condition (Low screen VR vs. other conditions; p < 0.05).

^b^ significant age*

condition effects (High & Low screen VR vs. Low Room and No VR; p < 0.05).

A significant age*condition interaction for transformation of time, F (2,26) = 2.30, p < 0.01, ES = 0.30, indicated that transformation of time was significantly higher for the middle-aged group in the high VR (3.47) and low-screen (3.94) conditions (p’s < 0.05) compared to the low-room and No VR conditions. No further main or interaction effects were recorded (p’s > 0.05).

## Discussion

The current study compared exercise performance and perceptions (enjoyment and engagement) in younger, middle-aged, and older adults during cycling exercise at two exercise intensities beneficial for health (moderate; steady state and high intensity (HIT) training; sprints) under different levels of VR immersion (High HMD, Low Room, Low Screen and No VR). In partial support of our hypothesis, exercise intensity had a significant effect of age on heart rate (steady state and sprint), cadence (sprint) and distance (sprint). Significant condition effects were observed during the sprint exercise for heart rate. No other significant condition effects were observed for exercise performance. Significant condition effects were identified for exercise engagement relating to the highest sense of control and loss of self-consciousness occurring in the Low Screen condition for all age groups, while transformation of time was significantly different between conditions in the middle-aged adult group (highest during High VR and Low Screen conditions). These results indicate that irrespective of age, participants found themselves in control and immersed more during the Low VR conditions than the High VR and No VR conditions.

### Exercise performance

The participants worked at similar exercise intensities across all conditions. This is no surprise as the participants were instructed to maintain 60% and 75–85% MHR during the steady state and sprint sessions, respectively. However, during the sprint cycling session, we observed lower HR in the high VR condition (although still within the prescribed range of MHR). The lower HR reflects a reduced physiological load in the high VR compared to low and No VR conditions. However, no reduction in perceived effort (i.e., RPE) or power output was recorded. Therefore, despite a lower HR, sprint cycling in the high VR condition did not appear to be perceived as less effort or impact exercise performance.

In terms of age-related effects on exercise intensity, as would be expected, the younger group worked at a higher HR compared to the middle and older aged groups. During the sprint cycling sessions, the older adults covered significantly less distance and had a lower cadence compared to the middle-aged and younger adults. This may be due to the older participants being more cautious in the high VR condition due to the HMD obscuring their normal vision and altering their balance ability (this will be discussed further later in the study), as well as age-related effects upon performance [34].

### Exercise perception (RPE, enjoyment and engagement)

Despite previous studies showing that exercise in VR can lower RPE [11] it is likely that the restrictive (as opposed to more autonomous) protocol used in this study to maintain the target intensities, precluded a potential difference in RPE. However, exercise enjoyment was found to increase as immersive level increased, a trend observed when all age groups were combined (see Table 4), yet differences between the VR conditions were not statistically significant. Despite not reaching statistical significance, age group trends indicated that the younger age group enjoyed the higher levels of immersion compared to the middle and older age groups who enjoyed the low level of immersion environments. This trend may be related to younger populations experiencing interactive gaming technology more, so being more familiar with this environment or being more open to these experiences than the older age groups [9]. Issues with balance and motion-sickness while wearing the HMD may also have hindered the older aged groups exercise experience in the high immersive condition. In line with previous research, these results imply that low immersion VR technology (e.g. Zwift, screen projection) could be important in establishing healthy exercise regimes for middle and older aged populations by enhancing exercise enjoyment [35].

Exercise engagement was measured using the Flow State Scale. Irrespective of age, during low screen VR immersion, adults reported feeling more in control and less self-conscious. This supports previous exergaming literature [22, 23] which found similar results for both control and loss of self-consciousness when participants played commercial exergames using the Nintendo Wii and XBOX Kinect. A potential reason we may have seen higher mean values occurring for the low-screen condition is due to the computer software used, Zwift being commercially available which may have meant that some participants may have experienced this platform previously. Although not significant, we observed lower mean scores for sense of control and loss of self-conscious and the higher mean scores for concentration on task during the High VR immersive environment. This could be due to the fact participants were using the HMD, which is fully immersive and therefore may make individuals less aware of their environment (i.e., more conscious of being unsteady on the bike or others in the room; see discussion below). In respect to unambiguous feedback, the high VR and low-room VR scored the lowest. This supports earlier work by Arndt et al. [31] who reported that experienced rowers had negative perceptions of a VR HMD indoor rowing environment over a traditional indoor rowing environment due to the lack of performance feedback available when wearing the HMD [22, 23].

The only age by condition interaction effect from the Flow State Scale data was for transformation of time (altered perception of time; either speeding up or down). The middle-aged group felt that time was altered during the High VR and Low Screen VR conditions compared to the No VR and Low Room VR conditions, whereas results were similar between conditions for the younger and older adults. From our data it is unclear if time sped up or slowed down. Previous findings [36] have shown that an individual’s perception of time is altered by exercise demand, of which our data suggest an age effect. Further research should explore whether participants perceive the exercise duration time to speed up or slow down depending on the immersion level and how age and/or experience may influence the direction.

The Flow State Scale results (see Table 5) show that irrespective of age, the low-screen VR environment provided the highest mean scores for sense of control, loss of self-conscious and transformation of time indicating that the task goals were clearly set and were achievable in respect to control and that the environment was immersive. This supports work by Robinson et al. [23] who found during exergaming with people with Multiple Sclerosis that those in the exergaming (Nintento Wii) group had significantly higher transformation of time scores compared to traditional balance exercise (no VR). Alongside the favourable trends towards greater enjoyment in the low immersive environments, this finding has implications for the use of low immersive VR to positively alter the exercise experience.

The exercise engagement results indicated lower scores on perceived ‘challenge-skill’ balance in the high VR environment (p > 0.05) indicating that participants may not have been able to get used to the HMD movements. During the HMD condition the riders had to lean at the torso when approaching bends and this was unfamiliar to many participants. Also bearing in mind the weight of the headset and restricted view of the real bike and surroundings, this unfamiliarity of the exercise situation potentially made the participants feel that they did not have the skill set to complete the challenge presented. In future, more time for participants to become familiar with the technology could be incorporated into the exercise protocol to see if this perception could be overcome. In addition, a small number of the participants (less than 5) reported minimal levels of dizziness and nausea during the HMD (high immersive) condition similar to previous studies that have investigated the use of HMDs [16, 24]. Interestingly this seemed to affect the middle age group the most and is possibly the reason why enjoyment was lower in this group. Future research in this area should include the simulator sickness questionnaire (SSQ) [37] to quantify the effects of cyber sickness on exercise enjoyment. The older age group experienced more balance maintenance issues during the HMD condition than nausea symptoms. This could be due to the continually moving image in the HMD and these participants being more cautious while exercising with the HMD on, this is supported by this age group having a lower HR but same RPE values in the high VR compared to the other VR conditions. Despite these challenges bought on by the high immersive conditions, the older group still reported these conditions as more favourable than the non-virtual environment. This suggests the increased distraction of the immersive conditions (away from the thought that they are exercising) could be useful in promoting healthy exercise habits in this population.

### Limitations and future research

This study examined exercise performance and perceptions (enjoyment and engagement measured) during VR immersion in younger, middle-aged, and older adults during cycling exercise. The mechanisms of the changes observed in exercise engagement remain to be determined. In addition to age, gender preferences between the differing virtual immersion levels or environments should be examined. Plante et al. [38] reported that females reported greater psychological benefits from combining VR with exercise than males. The authors found that even hours after the exercise, females reported enhanced energy and reduced tiredness levels following the VR exercise experience. Plante et al. [38] suggested this was due to the novelty of the VR experiences for females, as males are typically more likely to have prior experience of VR or similar gaming platforms [39]. Future research should look to establish whether gender differences (or psycho-social factors) influence exercise preferences (i.e., engagement and enjoyment) or when exercising in VR environments. In the current study, previous VR experience was not monitored, it may be that individuals with greater experience of VR environment where less affected by the differing environments or had become desensitised to the VR immersion of the HMD environment. Future research should consider monitoring VR experience when recruiting for further studies in this area. In the current study, one participant could not continue with the High VR environment due to high levels of nausea bought on by the visualisations. A small number of participants (<5) reported mild symptoms of dizziness and nausea during the High VR cycling environment but not enough to withdraw from the study. These symptoms may have had an effect on the individual’s level of enjoyment during this condition but without quantifying the extent of symptoms we are unable to make any further interpretation of its possible effect on the findings of the current study. Future research should also look to include the simulator sickness questionnaire to quantify any possible effects on exercise performance and engagement.

## Conclusion

The study compared exercise performance and perceptions (enjoyment and engagement) in younger, middle-aged, and older adults during cycling exercise at two different intensities beneficial for health (moderate and high intensity (HIT) training) under different levels of VR immersion (none, low and high). The findings revealed that VR can alter engagement (e.g., sense of control, loss of self-consciousness and transformation of time) and potentially exercise enjoyment for people aged 18–69 years. This study has identified some age group specific effects and preferences which should be considered when developing a physical activity exercise programme. For example, some populations may find the high immersive VR resulting in nausea and balance control issues. In this study, the use of a computer screen (displaying a virtual cycling environment responding to participant’s cycling efforts) placed in front of an exercise bike (low immersion) appeared to be well-tolerated for all age groups and resulted in the most favourable exercise enjoyment and engagement scores. This study supports the application of exercise in low immersive VR environments to promote more physically active lifestyles in all age groups. More research is needed to determine whether the use of high immersive environments could be beneficial to promote physically activity lifestyles.
